# Kynurenine: an oncometabolite in colon cancer

**DOI:** 10.15698/cst2020.01.210

**Published:** 2020-01-03

**Authors:** Niranjan Venkateswaran, Maralice Conacci-Sorrell

**Affiliations:** 1 Department of Cell Biology, University of Texas Southwestern Medical Center, Dallas, Texas 75390, USA.; 2 Harold C. Simmons Comprehensive Cancer Center, University of Texas Southwestern Medical Center, Dallas, Texas 75390, USA.; 3 Center for Regenerative Science and Medicine, University of Texas Southwestern Medical Center, Dallas, TX, 75390, USA.

**Keywords:** tryptophan, kynurenine, IDO1, TDO2, colon cancer, MYC, AHR

## Abstract

Tryptophan is one of the eight essential amino acids that must be obtained from the diet. Interestingly, tryptophan is the least abundant amino acid in most proteins, a large portion of cellular tryptophan is converted into metabolites of the serotonin and kynurenine pathways. In a recent study, (Venkateswaran, Lafita-Navarro et al., 2019, Genes Dev), we discovered that colon cancer cells display greater uptake and processing of tryptophan than normal colonic cells and tissues. This process is mediated by the oncogenic transcription factor MYC that promotes the expression of the tryptophan importers SLC1A5 and SLC7A5 and the tryptophan metabolizing enzyme AFMID. The metabolism of tryptophan in colon cancer cells generates kynurenine, a biologically active metabolite necessary to maintain continuous cell proliferation. Our results indicate that kynurenine functions as an oncometabolite, at least in part, by activating the transcription factor AHR, which then regulates growth promoting genes in cancer cells. We propose that blocking kynurenine production or activity can be an efficient approach to specifically limit the growth of colon cancer cells. Here, we describe our findings and new questions for future studies targeted at understanding AHR-independent function of kynurenine, as well as interfering with the enzyme AFMID as a new strategy to target the kynurenine pathway.

## TRYPTOPHAN IS PREFERENTIALLY CONVERTED INTO KYNURENINE IN COLON CANCER

**Figure 1:**
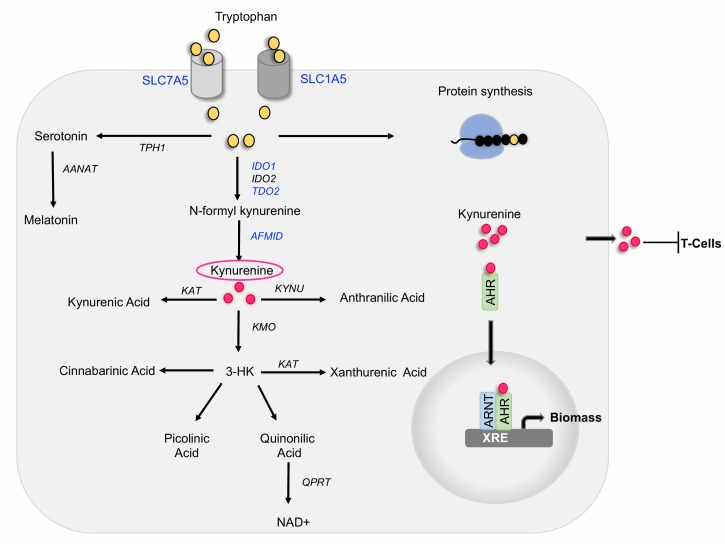
FIGURE 1: Summary of major routes for tryptophan uptake and utilization. Tryptophan is transported by the solute carriers SLC1A5 and SLC7A5. Intracellular tryptophan can be incorporated into proteins or metabolized by the serotonin or the kynurenine pathways. Proteins labelled in blue are transcriptionally upregulated by MYC and as consequence are highly expressed in colon cancer cells. Kynurenine can be exported by cancer cells and cause T-cell inactivation. Intracellular kynurenine binds to and activates the transcription factor AHR, leading to its nuclear translocation. In the nucleus AHR heterodimerizes with ARNT to promote the expression of genes that contain xenobiotic responsive elements (XRE) in their promoters, including genes involved in xenobiotic response and a signature of genes that promote protein synthesis.

Tryptophan can be utilized in three ways intracellularly: it can be incorporated into newly synthesized proteins, it can be processed by the serotonin pathway to give rise to serotonin and melatonin, and it can be processed by the kynurenine pathway to generate multiple biologically active catabolites **(Figure 1)**. The kynurenine pathway is aberrantly activated in cancer cells in a process that we discovered to be regulated by the activity of the oncogene MYC.

In the first step of the kynurenine pathway, tryptophan is metabolized by one of the three functionally equivalent enzymes: indoleamine 2,3-dioxygenase1 (IDO1), indoleamine 2,3-dioxygenase2 (IDO2), or tryptophan 2,3-dioxygenase 2 (TDO2) to produce N-formyl kynurenine, which is then converted into kynurenine by the activity of arylformamidase (AFMID) **(Figure 1)**. Kynurenine can be converted into kynurenic acid through the activity of kynurenine aminotransferase (KAT), into 3-hydroxy kynurenine by kynurenine 3-monooxygenase (KMO) or into anthranilic acid by kynureninase (KYNU). 3-hydroxy kynurenine is further metabolized into cinnabarinic acid, picolinic acid, or quinolinic acid, which gives rise to nicotinamide adenine dinucleotide (NAD), a co-enzyme that mediates redox reactions in a number of metabolic pathways, including glycolysis. While the liver contains all the enzymes necessary to metabolize tryptophan via the kynurenine pathway to generate NAD, the expression of specific enzymes by other tissues determine which tryptophan metabolites those tissues generate.

We found that samples of colon cancer have higher kynurenine levels than the respective matched healthy colon samples. This elevation in kynurenine correlates with the expression of the enzymes TDO2, IDO1, and AFMID in the same patient samples. Interestingly, TCGA analysis demonstrated that most colon cancer samples have greater expression of the tryptophan transporters SLC1A5 and SLC7A5 and the enzyme AFMID than the normal tissues from the same patients. The levels of TDO2 and IDO1 are elevated in fewer patients. Importantly, enzymes involved in further degrading kynurenine into downstream catabolites are not upregulated in the colon cancer samples, thus suggesting that kynurenine is a predominant metabolite of this pathway in colon cancer cells. Indeed, our results show that kynurenine is present at equimolar concentrations with tryptophan in colon cancer samples. All other catabolites are present at levels orders of magnitude lower. These results led to the hypothesis that kynurenine is biologically active in colon cancer.

## KYNURENINE FUNCTIONS AS AN ONCOMETABOLITE

Extensive research has established that tumor-produced kynurenine is exported into the tumor microenvironment, where it causes T-cell inactivation. This leads to immune evasion and survival of cancer cells. In addition to eliciting a paracrine effect on T-cells, our work, together with a growing body of data from others, established that kynurenine promotes growth of cancer cells by cell-autonomous mechanisms. Molecularly, the most well-understood function of kynurenine is as a ligand for the transcription factor aryl hydrocarbon receptor (AHR). Our lab has previously shown that AHR expression is elevated in colon tumors where it drives the expression of genes necessary for cell growth. Our recent study demonstrated that kynurenine is co-induced with AHR in the same tumors, thus indicating that the kynurenine-AHR pathway is active in colon cancer cells.

Our work demonstrated that blocking the conversion of tryptophan into kynurenine is an effective strategy to specifically limit the proliferation of colon cancer cells and transformed colonic organoids. Interestingly, blocking the binding of kynurenine to AHR also reduces the proliferation of colon cancer cells, however, less dramatically than inhibiting kynurenine production. Our results suggest that AHR-independent functions of kynurenine may exist. Future studies comparing cellular functions of kynurenine in AHR-deficient and wild-type cells will provide information on novel and potentially critical functions of kynurenine as an oncometabolite.

## MYC REGULATES TRYPTOPHAN UPTAKE AND ITS CONVERSION INTO KYNURENINE

The transcription factor MYC is a potent oncogene, which is upregulated in the majority of human tumors. MYC promotes cellular transformation by driving metabolic reprograming that leads to biomass accumulation and cellular proliferation. Using a combination of approaches, we discovered that MYC-transformed cells exhibit an increase in tryptophan uptake and processing by the kynurenine pathway. We found that MYC promotes the import of tryptophan into colon cancer cells by transcriptionally driving the expression of SLC7A5 and SLC1A5, which are capable of transporting tryptophan. MYC also induces the expression of the enzyme arylformamidase (AFMID) in cultured colon cancer cells. Using ^13^C-tryptophan and mass spectrometry, we found that the conversion of tryptophan into kynurenine was greater in MYC-driven colon cancer cells than normal primary colonic epithelial cells.

Our study demonstrates for the first time that a specific oncogenic hit directly drives kynurenine production and that transformed cells require kynurenine for growth. An important question that remains unanswered is whether or not kynurenine is necessary for tumor initiation and whether altered tryptophan uptake and metabolism precedes the formation of cancerous lesions. Because MYC was shown to be indispensable for colon cancer initiation in animal models and because MYC expression is elevated in almost all colon cancers samples, we predict that kynurenine levels will be globally elevated in colon cancer patients. Sensitive methods to detect kynurenine in the plasma could become an efficient non-invasive approach to identify patients with increased risk of colon cancer.

## TARGETING TRYPTOPHAN METABOLISM TO TREAT CANCER

The observation that IDO1 is elevated in several tumors spurred the development of inhibitors of IDO1 with the goal of inhibiting kynurenine production to allow the clearance of cancer cells by the patient's own T-cells. Unfortunately, clinical trials using IDO1 inhibitors to treat various types of cancer have been unsuccessful, potentially due to redundant functions played by IDO2 and TDO2. Our results regarding the importance of AFMID in the kynurenine pathway in colon cancer open the exciting possibility that AFMID may provide a better target to abrogate kynurenine production *in vivo*. Nonetheless, additional studies are necessary to better define the contribution of AFMID to cellular growth and to determine whether this enzyme can be targeted to limit kynurenine production and prevent cancer cell growth without affecting the integrity of normal tissues.

Future studies targeted at defining AHR-independent roles of kynurenine and its catabolites could be key to fully comprehend the effects of the kynurenine pathway in cancer. One or more tryptophan catabolites could provide diagnostics or prognostics tools for colon cancer and other diseases. Implementing sensitive methods to quantify tryptophan uptake and processing *in vivo* will lead to the identification of tumors that are dependent on tryptophan and kynurenine, thus defining a patient population that have the potential of responding to inhibition of the kynurenine pathway.

